# Differential *in vitro* and *in vivo* responses of *Akkermansia muciniphila* to *Odontosoria biflora* (Kaulf.) C.Chr. [*Lindsaeaceae*] hexane extract in diet- and alloxan-induced BALB/c mice

**DOI:** 10.3389/abp.2026.16199

**Published:** 2026-02-24

**Authors:** Marvie Hamel Darbandi, Leslie Michelle M. Dalmacio

**Affiliations:** 1 Department of Biochemistry and Molecular Biology, College of Medicine, University of the Philippines Manila, Metro Manila, Philippines; 2 Department of Biochemistry, Nutrition and Molecular Biology, School of Medicine, Bohol Island State University, Tagbilaran, Bohol, Philippines

**Keywords:** *Akkermansia muiciniphila*, BALB/c mice, diabetes mellitus, fecal qPCR, *in vitro* and *in vivo* comparison

## Abstract

*Akkermansia muciniphila* is a mucin-degrading gut bacterium linked to metabolic health, yet culture-based growth stimulation may not translate to sustained enrichment *in vivo*. *Odontosoria biflora* (“tubho”) is traditionally consumed in the Philippines as a herbal tea, but its extract-level activity toward *A. muciniphila* remains poorly characterized. *O. biflora* was sequentially extracted (hexane, ethyl acetate, methanol, aqueous) and screened for growth-supporting activity toward *A. muciniphila* in modified BHI under anaerobic conditions. The most active fraction (hexane; OBE HEX) was evaluated for acute oral tolerability in BALB/c mice according to OECD Tesy No. 423 (up to 2000 mg/kg) and subsequently assessed in a high-fat/high-sugar diet plus alloxan-induced diabetic model. Fecal *A. muciniphila-*specific qPCR signal was monitored at weeks 0, 1, 2, and 4 using a modified 2^−^ΔΔCt approach with external ATCC genomic DNA as a reference. OBE-HEX produced the strongest *in vitro* growth-supporting effect (56.43% at 250 mg/L, p < 0.05; 85.62% at 500 mg/L, p < 0.001) and showed no observable toxicity *in vivo*. In contrast, *in vivo* analysis revealed only transient changes in fecal *A. muciniphila* detection following OBE-HEX administration, whereas sustained elevation was observed only in metformin-treated mice. Untargeted UPLC-ESI-QTOF-MS analysis of OBE-HEX yielded putative identification of 2-O-rhamnosylvitexin and 7-methoxy-9,10-dihydrophenanthrene-2,5-diol. Overall, these findings demonstrate that while *O. biflora* hexane extract exhibits direct growth-supporting activity toward *A. muciniphila in vitro* and is orally tolerable, such effects do not translate into sustained *in vivo* enrichment under diabetic conditions, underscoring the limitations of extrapolating culture-based microbiota screening results to host-associated systems.

## Introduction

Type 2 diabetes mellitus (T2DM) is the most common form of diabetes and is characterized by chronic hyperglycemia driven by insulin resistance and progressive beta cell dysfunction (American Diabetes Association Professional Practice Committee, 2024; Galicia Garcia et al., 2020). In the Philippines, cohort evidence indicates a sustained burden of T2DM among adults (Soria et al., 2009). Beyond its clinical relevance, T2DM is frequently employed as an experimental model of metabolic and inflammatory stress that disrupts host–microbe interactions and intestinal homeostasis (Chong et al., 2025; Fliegerová et al., 2025).

The gut microbiota is increasingly viewed as a mechanistic interface linking diet, immunity, and host metabolism. Gut dysbiosis and functional microbial shifts are associated with impaired glucose regulation, compromised intestinal barrier integrity, and metabolic endotoxemia, processes that can amplify low grade inflammation and insulin resistance in T2DM (Gurung et al., 2020; Crudele et al., 2023; An et al., 2022). Accordingly, diet-derived microbiota interacting factors have been investigated as tools to probe species-specific microbial responses under perturbed metabolic conditions rather than solely as therapeutic interventions (Sahhaf Ebrahimi et al., 2019; Wang et al., 2024).

Among gut-associated bacteria, *Akkermansia muciniphila* has gained particular attention as a novel probiotic candidate; its abundance is consistently linked to improved glucose homeostasis, making it a key microbial marker for metabolic health in T2DM (Rodrigues et al., 2022; Dianti et al., 2022). Clinical and experimental studies report associations between *A. muciniphila* abundance and improved insulin sensitivity in T2DM, including evidence from pasteurized *A. muciniphila* supplementation trials (Depommier et al., 2019). Importantly, *A. muciniphila* was selected in the present study as a biologically informative taxon to assess species-specific microbial responses, rather than as a representative of probiotic bacteria as a whole.

Plant based foods and medicinal plants provide diverse compounds that may modulate gut bacteria. Beyond classical prebiotic fibers, polyphenols and other secondary metabolites can influence bacteria directly or indirectly through altered mucin metabolism, microbial cross feeding, redox balance, and intestinal barrier function (Rodríguez-Daza and de Vos, 2022; Barik et al., 2024; Xu et al., 2019). However, culture-based growth stimulation does not guarantee enrichment *in vivo*, where host physiology and microbial competition constrain ecological outcomes.

*Odontosoria biflora*, commonly known as “tubho” fern, is traditionally consumed as an herbal tea by the Ivatan people of the Philippines and is culturally regarded to have antioxidant property, medicinal benefits and longevity (Slow Food Foundation for Biodiversity, 2018; Department of Science and Technology – Provincial Science and Technology Office Region 2, 2024). To date, this is the first experimental investigation of *O. biflora* potential bioactivity and interaction with gut-associated bacteria. While the whole plant contains diverse nutritional components, including fiber and carbohydrates, the present study focuses on solvent extracts to evaluate extract-level bioactivity rather than whole plant dietary effects. Related *Odontosoria* species are rich in bioactive phenolics, tannins, and flavonoids known to inhibit carbohydrate-hydrolyzing enzymes, a mechanism relevant to glycemic regulation (Johnson et al., 2020; Rohn et al., 2002). The presence of flavonoid derivatives like quercetin glycosides provides a biochemical basis for exploring potential interactions with *A*. *muciniphila*, a mucin-associated bacterium known to respond to polyphenolic environments (Tang et al., 2024).

In this study, *A. muciniphila* was selected as a biologically informative taxon to evaluate microbiota-related responses under controlled *in vitro* and *in vivo* conditions. Sequential extracts of *O. biflora* were screened for growth-supporting activity toward *A. muciniphila in vitro*, and the most active fraction was subsequently evaluated for oral tolerability following OECD guidelines (OECD Test No. 423, 2002). Furthermore, *in vivo* effects on fecal *A. muciniphila*-specific qPCR signal were assessed using a high-fat/high-sugar diet combined with an alloxan-induced diabetes model, which integrates insulin resistance with beta-cell injury (Ighodaro et al., 2017; Al-Awar et al., 2016; Binh et al., 2021).

## Materials and methods

### Sample collection and preparation of *O. biflora* extraction

Five hundred grams of *O. biflora* (Kaulf.) C. Chr. [Lindsaeaceae] leaves and stem were collected from Chavakan, Sinakan Sabtang, Batanes Island, Philippines were harvested, sectioned, thoroughly rinsed with distilled aqueous solution, and allowed to air-dry for 48 h. Botanical authentication of the plant material was conducted at the Herbarium of the Institute of Biology, University of the Philippines Diliman. The dried samples were subsequently cut into uniform strips, immersed, and stored in airtight containers prior to extraction. Sequential solvent extraction was performed using solvents of increasing polarity, namely hexane, ethyl acetate, methanol, and an aqueous solvent (Hamel Darbandi et al., 2026). The collected extracts from one solvent were pooled and subjected to a rotary evaporator (Büchi, Switzerland) until completely dry.

### Determination of the effect of the different extracts of *O. biflora* on the growth of *A. muciniphila in vitro*

To assess the growth response of *A. muciniphila, Odontoria biflora* extracts (OBE) were prepared through sequential solvent extraction using hexane, ethyl acetate, methonal and aqueous. The most active extract was then utilized in the subsequent experiments. Briefy, the *A. muciniphila* type strain Muc^T^ (= ATCC BAA-835^T^ = CIP 107961^T^), obtained from the Japan Collection of Microorganisms (JCM) at the RIKEN BioResource Research Center, was cultured anaerobically in Brain Heart Infusion (BHI) medium, following the protocol of Lou et al. (2021) with modifications. To evaluate the growth-promoting activity of OBE, Brain Heart Infusion (BHI) broth was supplemented with OBE at final concentrations of 250 mg/L and 500 mg/L, along with 2 g/L glucose as an additional carbon source. These concentrations were selected based on established extract supplementation ranges reported in microbial growth assays, including Xia et al. (2021), who supplemented cultures with bioactive plant compounds at comparable upper limits (150–500 mg/L), demonstrating the feasibility of high-dose phytochemical testing in *A. muciniphila* growth. Cultures were incubated anaerobically at 37 °C for 24 h, consistent with the short-term growth and metabolic response assays reported for *A. muciniphila* (Xia et al., 2021; Liu et al., 2024). Anaerobic conditions were generated using the candle-jar method of Saha et al. (2016), which provides a cost-efficient low-oxygen environment suitable for anaerobe cultivation.

The relative growth yield of *A. muciniphila* was determined by measuring optical density at 620 nm and applying the modified calculation of Xia et al. (2021), where A represents the OD_620_ of cultures grown in BHI with glucose and OBE, and B represents the OD_620_ change of cultures grown with BHI and OBE alone. Relative growth was computed using the formula:

$$\text{Relative growth}=\frac{A-B}{B}\times \;100\%$$

The experiment was conducted in three independent trials, each performed in triplicate.

### Determination of the acute oral toxicity assay

The study design followed the general principles of internationally accepted acute oral toxicity guidelines for rodents, and the results were used to establish the safe dose range for subsequent *in vivo* assays. Acute oral toxicity of *Odontosoria biflora* hexane extract (OBE HEX) was evaluated in male BALB/c mice (6–7 weeks, 20–25 g) sourced from IACUC approved and Bureau of Animal Industry (BAI) accredited breeder (Mots Animal House Laboratory and Research, Sta. Rosa city, Laguna). Four mice received single oral doses of 250, 500, 1,000, or 2000 mg/kg (1 mouse per dose). OBE HEX was formulated in 1 percent dimethyl sulfoxide (DMSO) in normal saline, a vehicle concentration considered safe for murine studies (Tomislav et al., 2011). Clinical signs, including changes in skin and fur, eyes and mucous membranes, respiration, circulation, autonomic and central nervous function, behavioral activity, and specific indicators such as gastrointestinal discomfort, diarrheal spots, tremors, convulsions, lethargy, or coma, were recorded twice on Day 1 and once daily thereafter for 14 days in accordance with internationally accepted acute oral toxicity guidelines for rodents (Organization for Economic Co-operation and Development [OECD], 2002). Each mouse was classified as A (death), B (evident toxicity), or C (no toxicity) (OECD Test No. 423, 2002).

### Animal model

**FIGURE 1:**
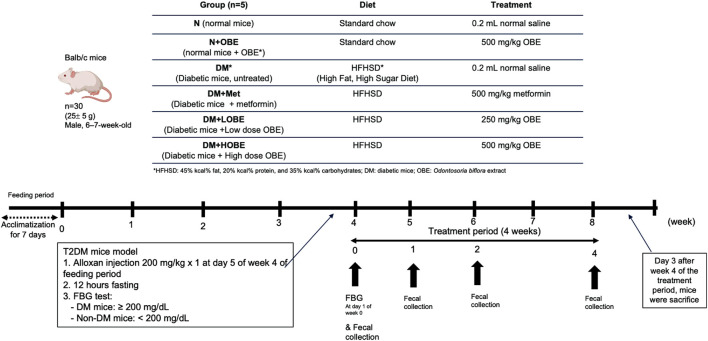
Schematic diagram of animal set-up model.

Thirty males, 6–7-week-old BALB/c mice (25 ± 5 g) were sourced from Bureau of Animal Industry (BAI) accredited breeder, and provided with a standard diet and free access to water. The mice were maintained at 20 °C–22 °C, with five mice per cage and housed in the Multidisciplinary Laboratory, Paz Mendoza Building, University of the Philippines Manila. The mice (n = 30) were divided into six groups (Figure 1): 1) N, normal mice receiving 0.2 mL normal saline; 2) N + OBE, normal mice administered with 500 mg/kg OBE; 3) DM, untreated diabetic mice; 4) DM + MET, diabetic mice treated with 500 mg/kg metformin; 5) DM + LOBE, diabetic mice treated with 250 mg/kg OBE (low dose); and 6) DM + HOBE, diabetic mice treated with 500 mg/kg (high dose). The doses of OBE and metformin were selected based on established dosing ranges for plant-derived extracts in BALB/c mouse models. Metformin was administered at 500 mg/kg as a reference treatment (Aslam et al., 2017) while OBE was given at 250 mg/kg and 500 mg/kg, doses commonly used in BALB/c studies evaluating biological responses to whole-plant extracts (Qin et al., 2022; Ali et al., 2021). Dose selection was further supported by acute oral toxicity assay conducted in the present study.

After a 1-week acclimatization period, the N and N + OBE groups were fed with standard chow diet (Altromin 1324, Altromin GmbH, Lage, Germany), while all DM groups were fed a high-fat, high-sugar diet (Research Diets D12451, New Brunswick, NJ, USA), containing 45% kcal% fat, 20% kcal% protein, and 35% kcal% carbohydrates for 8 weeks. On day 5 of week 4, all DM groups were injected intraperitoneally with a single dose of 200 mg/kg alloxan (Sigma Aldrich, UK) to induce diabetes (Aslam et al., 2017).

Alloxan has long been used as a standard procedure to induce hyperglycemia in experimental diabetes models across various species (Ighodaro et al., 2017). While alloxan typically produces an insulin-deficient phenotype, pairing it with a high-fat/high-glucose diet can yield a type-2-like state characterized by insulin resistance plus β-cell dysfunction, as demonstrated in murine models for type 2 diabetes mellitus (Binh et al., 2021; Bondar et al., 2025). After 12 h of fasting, fasting blood glucose (FBG) was measured using Lifescan ONETOUCH UltraPlus glucometer (Johnson & Johnson, New Jersey, US), the glucometer measures glucose in a whole blood sample, but it is plasma-calibrated so that the results are displayed as plasma-equivalent values. This is done to allow for easier comparison with laboratory test results, which are typically reported in plasma-equivalent units. Diabetic mice are described as having a blood glucose level of more than 200 mg/dL, while non-diabetic mice are described as having a blood glucose level lower than 200 mg/dL (Aslam et al., 2017). The most active extract of OBE obtained from *in vitro* studies were used as treatment and administered via oral gavage every other day for 4 weeks of the treatment period. The protocols were submitted and approved by the Institutional Animal Care and Use Committee (IACUC Protocol No. 2024-003) of University of the Philippines Manila and performed completely in line with the guidelines of the Animal Ethics Committee. Mice were sacrificed on the 3rd day after week 4 of the treatment period through cervical dislocation as a euthanasia procedure approved by the UP Manila IACUC.

### Determination of the effects of OBE in *Akkermansia muciniphila*

#### Fecal sample collection

Fecal samples from each mouse were collected at weeks 0, 1, 2, and 4 of the treatment period. Since gut microflora from the distal gut are shed from feces, fecal samples were collected directly from the mice or from the sterile beddings. Beddings were changed with new autoclaved beddings before collection and fecal samples collected from each mouse were stored in individual sterile 1.5 mL microcentrifuge tubes, accordingly labeled, at 4 °C.

#### Fecal DNA extraction and bacterial DNA quality and quantity assessment

Microbial DNA was extracted from 200 mg fecal samples of each mouse using the E. Z.N.A.® Stool DNA Kit (Omega Bio-Tek, Norcross, GA, United States), following the manufacturer’s protocol. A 200 mg of the fecal samples for each mouse and time point was added to the dry bead tube containing garnet beads. The mechanical collisions between the beads lysed the cells combined with chemical lysis by SLX-Mlus Buffer, ensuring efficient release of microbial DNA from fecal samples. To remove common substances in fecal samples that may interfere with the downstream analyses, the kit has a cationic High Throughput (cHTR) Reagent and pre-lysis (P2) Buffer. Total genomic DNA was captured on a silica spin column and was washed and eluted to a new collection tube. The bacterial genomic DNA was prepared for downstream applications including quantitative PCR and next-generation sequencing analysis. The extracted DNA concentration and purity were measured using the Thermo ScientificTM NanoDropTM Spectrophotometer 2000 (Wilmington, USA). Solution C6 or the elution buffer from the E.Z.N.A. Stool DNA Kit was used as blank. The dsDNA concentration was expressed in ng/µL and computed by multiplying the A260 reading with the dilution factor, and using the relationship that an A260 of 1.0 = 50 μg/mL pure dsDNA. Purity was measured by finding the ratio of the absorbance readings at 260/280 and 260/230.

#### Quantification of *A. muciniphila*

Relative quantification of *A. muciniphila* was done using GoTaq® qPCR Master Mix (Promega) with primer sequences detailed on Table 1 and sourced from Genomics Laboratory. The mastermix contained carboxy-X-rhodamine (BRYT) Green® Dye as the fluorescent dye and Hot Start polymerase together with the reaction buffer, MgCl_2_, dNTPs and stabilizers. Each sample was done in triplicate and a “no-template” negative control was included. The qPCR run consists of enzyme activation at 95 °C for 2 min, followed by 40 cycles of denaturation at 95 °C for 15s, annealing for 60s at 60 °C and extension at 72 °C for 60s, and a final extension for 5 min at 72 °C. A melt curve was generated afterwards to confirm the specificity of the reaction. Amplification and melting curves were analyzed using the Quantitation–Comparative Ct (ΔΔCt) and Tm analysis module of the CFX Opus 96 Real-Time PCR System (Bio-Rad Laboratories, Hercules, CA, USA). Relative quantification was performed using a modified 2^−ΔΔ^Ct approach in which genomic DNA from *A. muciniphila* ATCC strain served as the external reference template, and the Normal group acted as the biological calibrator. Validation of this approach was supported by single-peak melt curves, consistent Ct values for the ATCC reference across runs, and acceptable reproducibility among technical triplicates, indicating specific amplification and matched reaction efficiencies between reference and sample DNA. This modified comparative Ct approach is consistent with bacterial qPCR workflows that quantify species-specific targets relative to a reference template and compute fold-changes using the 2^−^ΔΔCt method, as demonstrated by Sezgin et al. (2022). Relative abundance was calculated according to Livak and Schmittgen (2001) using the equation:

$$\text{Relative expression}=2^{-\Delta \Delta Ct}$$

**TABLE 1:** *Akkermansia muciniphila* 16S RNA gene-based primer.

Bacterial target	Direction	Primer sequence (5’→3′)	Amplicon size
*Akkermansia muciniphila*	Forward Reverse	CAGCACGTGAAGGTGGGGAC CCTTGCGGTTGGCTTCAGAT	329 bp

Where:

Δ *Ct* = ^*Ct*^ target gene (sample) – Ct reference gene (*A. muciniphila* ATCC strain)
Δ Δ *Ct* = ^Δ^ ^*Ct*^ target gene (treatment group) – ^Δ^ ^*Ct*^ reference gene (normal control group)

### Determination of the chemical composition of the selected *O. biflora* extract and its proximate analysis

#### Metabolite profiling

Sample preparation was done by dissolving 10 mg of the extract in 200 µL of DMSO, followed by the addition of 4.8 mL of acetonitrile and then homogenized using a vortex mixer. Subsequently, the solution was filtered through a 0.2-µm PTFE syringe filter and transferred into clear LC-MS vials. Acetonitrile served as the blank for analysis, while quercetin was used as standard. Metabolite profiling was performed using high-resolution ultra-performance liquid chromatography coupled with electrospray ionization/quadrupole time-of-flight mass spectrometry (HR-UPLC-ESI-QTOF-MS) at University of the Philippines Los Baños. Chromatographic separation was done by a reverse-phase Waters ACQUITY HSS C18 column (2.1 mm × 100 mm, 1.8-µm particle size). To enhance mass accuracy, the mass analyzers were calibrated using a 0.5-mM sodium formate solution. A lock mass solution containing leucine-enkephalin at a concentration of 200 pg/μL in a 50:50 (v/v) acetonitrile–water mixture with 0.1% formic acid was continuously infused at 30-s intervals throughout the LC run, with each spray scanned for 1.5 s (m/z 556.2771). The mobile phase consisted of (A) ultrapure water with 0.1% formic acid and (B) acetonitrile with 0.1% formic acid. A gradient elution program was employed as follows: 15%–40% B (0–1.67 min), 40%–55% B (1.67–5.00 min), 55%–75% B (5.00–6.67 min), 75%–80% B (6.67–10.84 min), 80%–95% B (10.84–13.34 min), 95%–15% B (13.34–15.01 min), and finally, 15%–5% B (15.01–18.00 min). LC-MS data acquisition was performed using MassLynx 4.2 software, covering a mass range of 50–1,500 Da. Instrument settings included a cone voltage of 40 V, source offset of 80 V, capillary voltage of 3.0 kV, source temperature of 120 °C, and desolvation temperature of 450 °C. Nitrogen was used as the desolvation gas at a flow rate of 600 L/h, while argon was employed as the cone gas at 100 L/h. The column was maintained at 30 °C, and the sample storage temperature was set to 15 °C. Electrospray ionization was performed in positive ionization mode, utilizing the data-independent acquisition method (MSE mode) in continuum format. Collision energy settings included 6 eV for low-energy scans and a ramp from 30 to 50 eV for high-energy scans. The photodiode array (PDA) detector was configured to scan wavelengths ranging from 190 to 500 nm. The sample injection volume was 2 μL, with a flow rate of 0.25 mL/min. RAW files generated from the analysis were converted to ABF format using the Reifycs ABF Converter. Peak alignment, detection, and identification were processed using MS-DIAL version 4.9 software^1^, with spectral databases from the MS/MS-Pos libraries integrated within the software for compound identification. All the reagents used were sourced from Pascual Pharma Corp Laboratory (Philippines).

#### Proximate analysis

The ash, moisture, crude protein, crude fiber, total carbohydrate and total dietary fiber of *Odontosoria biflora* were outsourced in another institution (Department of Science and Technology-Industrial Technology Development Institute and National Institute of Molecular Biology and Biotechnology (BIOTECH), University of the Philippines, Los Baños.

##### Ash

The ash content of *O. biflora* was analyzed using the gravimetric method following AOAC Method 923.03 (modified, 21st ed.) (AOAC, 2019). About 2.0 g of the sample was placed in a porcelain crucible and heated in a furnace at 550 °C for 2 h. After cooling in a desiccator for 30 min, the crucible was weighed, and the final mass was recorded. The ash content was expressed as a percentage.

##### Moisture

The moisture content of *O. biflora* was measured using the air-oven method in accordance with AOAC Method 925.45B (modified, 21st ed.) (AOAC, 2019). About 2.0 g of the sample was placed in an aluminum dish and dried in an oven at 105 °C for 2 h. After cooling in a desiccator for 30 min, the dish was weighed, and the final mass was recorded. The moisture content was expressed as a % moisture.

##### Crude protein

The crude protein content was quantified using the Kjeldahl method, following AOAC Official Method 923.03 (modified, 21st ed.) (AOAC, 2019). The Kjeldahl nitrogen percentage and crude protein content were calculated using the formulas:

$$\text{Kjeldahl nitrogen},\%=\frac{\left(\text{Vs}-\text{VB}\right)\;\times \;M\;\times \;14.01\;}{W\;\times \;10}$$

Crude protein, % = % Kjeldahl Nitrogen × F (Conversion factor from nitrogen to protein)

Where Vs is the volume of standardized acid used to titrate the sample; VB is the volume of standardized acid used to titrate the reagent blank; M is the molarity of the standard HCl; 14.01 is the atomic weight of Nitrogen; 10 is the factor to convert mg/g to percent; and W is the weight of the sample.

##### Crude fiber

Crude fiber content was determined using the Kürschner–Hanak method, as described by Cvrk et al. (2022). From the homogenized bulk sample, 1.000 g was transferred into a 100-mL round-bottom flask, followed by the addition of 25 mL of 80% (v/v) acetic acid and 2.5 mL of concentrated nitric acid. The mixture was heated under reflux for 30 min, filtered hot through a G-3 sintered glass crucible, and the residue was sequentially washed with acetic–nitric acid mixture, hot water, ethanol, and diethyl ether. The residue was dried at 105 °C for 30 min, cooled in a desiccator, and weighed. Crude fiber content was calculated as:

$$\text{Crude fiber content }\left(\%\right)=\frac{a\times 100}{W}$$

where *a* a is the mass of the dried fiber residue (g) and *W* is the initial sample weight (g).

##### Total carbohydrate

Proximate parameters (carbohydrate, fats, protein and ash) of the plant *O. biflora* were determined using the Association of Official Analytical Chemists (AOAC, 2019) method. The nitrogen content of the samples was determined by the micro-Kjeldahl method. The nitrogen value obtained was multiplied by 6.25 to convert it to crude protein. The weight difference methods were used to determine moisture and ash content levels while crude fat of the plant was determined using the AOAC procedure with petroleum ether as solvent. The carbohydrate content was determined by calculation using the different method:

$$\%\text{Total Carbohydrate}=100-\%\;\left(\text{Protein}+\text{Fat}+\text{Moisture}+\text{Ash}+\text{Crude Fiber}\right)$$

##### Crude fat

The crude fat content of *O. biflora* was determined in accordance with AOAC Method 2003.05 (modified, 21st ed.) (AOAC, 2019). Approximately 2 g of the sample was placed into a tared extraction thimble and dried at 102 °C for 30 min. The thimble was then immersed in solvent and boiled for a minimum of 20 min, followed by extraction for 40 min. Afterward, the solvent was evaporated, and the extracted oil was quantified gravimetrically by difference in weight.

##### Dietary fiber

Dietary fiber analysis followed modified AOAC Official Method 991.43 (AOAC, 2019). A 1 g homogenized *O. biflora* sample was mixed with 40 mL MES-TRIS buffer (pH 8.2) and stirred until fully dispersed. After adding 50 µL alpha-amylase, the mixture was incubated at 95 °C–100 °C for 15 min with agitation, then cooled to 60 °C. Protease (10 µL) was added and incubated for 30 min at 60 °C. The pH was adjusted to 4.0–4.7 using 5 mL of 0.561N HCl and 1N NaOH, followed by the addition of 300 µL amyloglucosidase and incubation at 60.1 °C for 30 min. To precipitate dietary fiber, 225 mL of 95% ethanol was added at 60 °C, then the sample was left at room temperature for 1 hour. A Celite bed in a tared crucible was prepared with 15 mL of 78% ethanol, and vacuum filtration was performed. The enzyme digestate was filtered, and residues were washed twice with 78% ethanol, 95% ethanol, and acetone. The crucible was dried overnight at 105 °C, cooled in a desiccator for 1 hour, and weighed. The dietary fiber residue was calculated by subtracting the weight of the empty crucible containing only Celite.

### Statistical analysis

Statistical analysis for the *in vitro* bacterial growth assay was conducted using two-way ANOVA followed by Dunnett’s multiple comparison test. Quantitative PCR data were analyzed using two way RM-ANOVA followed by Dunnetts test. A p-value <0.05 was considered statistically significant. All statistical test were performed using GraphPad Prism version 10.5.0.

## Results

### Effect of the different extracts of O. biflora on the growth of A. muciniphila *in vitro*

**FIGURE 2:**
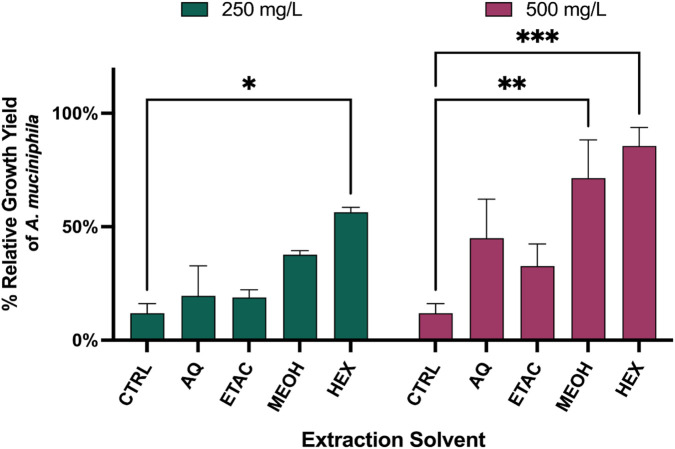
Effect of the different extracts of *O. biflora* on the growth of *A. muciniphila*. The percentage relative growth yield of *Akkermansia muciniphila* cultured in Brain-Heart Infusion (BHI) medium, supplemented with *Odontosoria biflora* extract (OBE) obtained using different extraction solvents, namely aqueous (AQ), ethyl acetate (ETAC), methanol (MEOH), and hexane (HEX), is presented. The bacterial strain was cultivated in BHI medium, with and without glucose-supplemented OBE, at final concentrations of 250 mg/mL and 500 mg/mL, and its growth was assessed via OD620 measurements. The experiment was conducted in three independent trials, each performed in triplicate. Statistical significance relative to the control is indicated by asterisks (p ≤ 0.05).

To identify the most active *O. biflora* extract on the growth of *A. muciniphila in vitro*, *O. biflora* extracts were evaluated using fractions obtained from sequential extraction, which are labeled in Figure 2 as AQ (*O. biflora* aqueous extract), ETAC (*O. biflora* ethyl acetate extract), MEOH (*O. biflora* methanolic extract), and HEX (*O. biflora* hexane extract). The bacterial strain was cultured in modified Brain-Heart Infusion (BHI) medium, with and without glucose-supplemented OBE, at 250 mg/L and 500 mg/L, and growth was assessed via OD620 measurements while *A. muciniphila* cultured in BHI and no OBE supplementation serve as the control (CTRL). At 250 mg/L, OBE-HEX increased *A. muciniphila* growth to 56.43% compared with 11.92% in the control (*p* < 0.05). At 500 mg/L, growth further increased to 85.62% (*p* < 0.001) (Figure 2). Therefore, the OBE HEX was used in the subsequent assays.

### Acute oral toxicity assay

All mice administered a single oral dose of *O*. *biflora* hexane extract (OBE-HEX) at 250, 500, 1,000, or 2000 mg/kg body weight survived and showed no treatment-related clinical signs, behavioral changes, or body-weight loss throughout the 14-day observation period. In accordance with OECD Test No. 423, the extract was well tolerated at the limit dose of 2000 mg/kg. This safety profile supported the selection of 250 mg/kg and 500 mg/kg as the low- and high-dose levels, respectively, for subsequent *in vivo* assays.

### Effect of OBE on fecal abundance of *Akkermanisia muciniphila*

**FIGURE 3:**
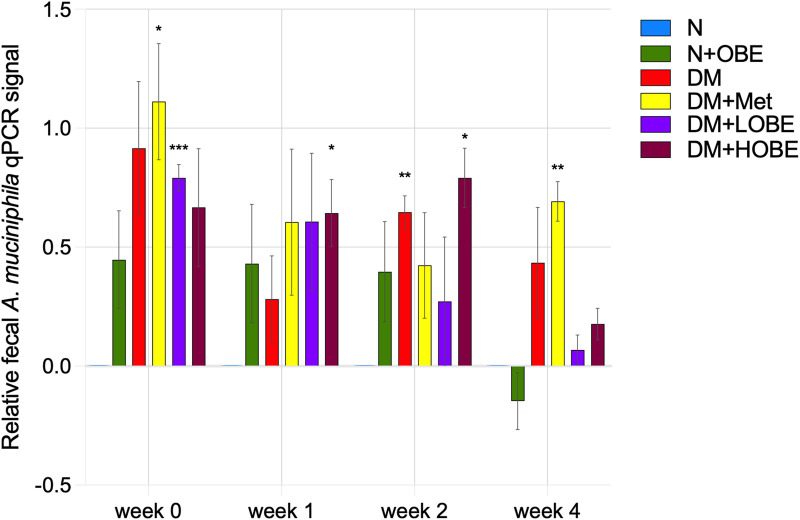
Relative *Akkermansia muciniphila*-specific signal in fecal samples of BALB/c mice. Relative detection levels were quantified using a modified 2^−^ΔΔCt approach with external *A. muciniphila* ATCC genomic DNA as reference and the normal group as biological calibrator. Data represented as mean ± SE. Statistical significance was determined by two-way repeated-measures ANOVA followed by Dunnett’s multiple comparisons test (*p* < 0.05, p < 0.01, *p* < 0.001).

Quantitative real-time PCR (qPCR) analysis targeting *A. muciniphila* was performed on fecal DNA to assess relative changes in *A. muciniphila–*specific qPCR signal across treatment groups and time points. At week 0, the DM + Met and DM + LOBE groups exhibited the highest relative detection level of *A. muciniphila* (p < 0.05 and p < 0.001, respectively) compared with the other groups. By week 1, a decline in relative qPCR signal was observed in the N + OBE, DM, DM + Met, and DM + LOBE groups, whereas the DM + HOBE group demonstrated a significant increase qPCR signal relative to N (p < 0.05). At week 2, both the DM (p < 0.01) and DM + HOBE (p < 0.05) groups showed marked increases in *A. muciniphila* relative qPCR signal, while DM + LOBE groups exhibited pronounced downregulation, resulting in the lowest values among all treatments. By week *4, A. muciniphila* levels had declined across most groups, with only the DM + Met group maintaining significantly elevated relative qPCR signal (p < 0.01). In contrast, the DM, DM + LOBE, and DM + HOBE groups displayed lower expression levels, and the N + OBE group showed the lowest detection overall (Figure 3).

### Metabolite profiling and proximate analysis of O.biflora

**FIGURE 4:**
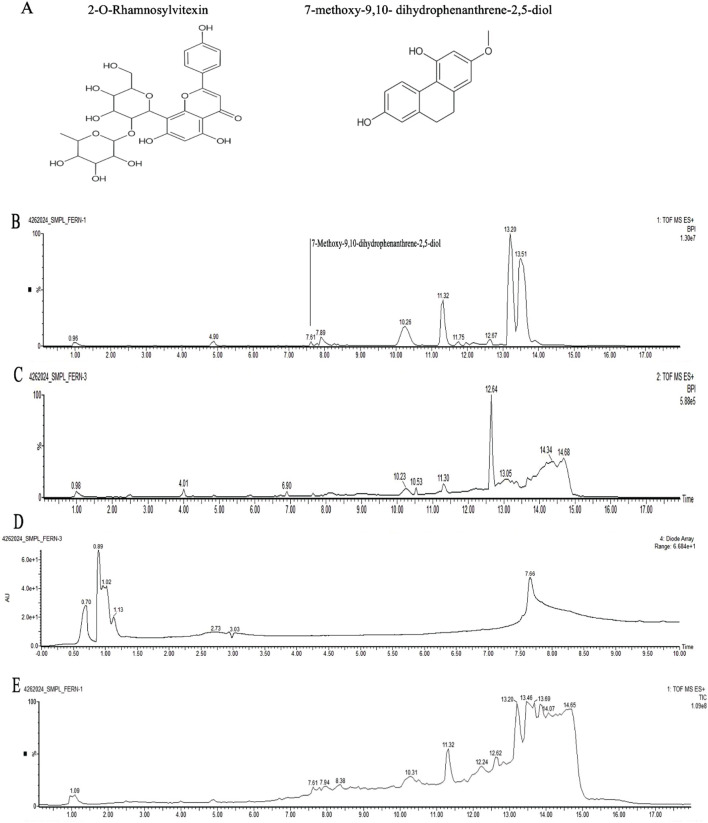
LC–MS/MS of hexane extract of *O. biflora*. **(A)** Chemical structure of 2-O-Rhamnosylvitexin and 7-methoxy-9,10- dihydrophenanthrene-2,5-diol **(B)** MS1Chromatograms Base Peak Intensities **(C)** MS2 Chromatograms Base Peak Intensities **(D)** PDA UV Chromatograms **(E)** Total Ion Chromatograms.

The metabolite profiling of OBE HEX was analyzed using high-resolution ultra-performance liquid chromatography coupled with electrospray ionization/quadrupole time-of-flight mass spectrometry (HR-UPLC-ESI-QTOF-MS) to obtain an untargeted overview of the extract constituents. Data acquisition was performed with MassLynx 4.2 software (mass range: 50–1,500 Da). RAW files were converted to ABF using Reifycs ABF Converter for further analysis. Peak alignment, detection, and identification were conducted using MS-DIAL (v4.9) with MS/MS-Pos libraries. Representative base peak ion chromatograms (BPI) from injections of each OBE extract (Figures 4B–E). Peak annotations correspond to the most intense retention time in each spectrum, representing distinct sample components.

Two metabolites were putatively identified in OBE HEX based on spectral similarity to reference library entries: 2-O-Rhamnosylvitexin (retention time 1.922 min, m/z 579.1639) and 7-methoxy-9,10-dihydrophenanthrene-2,5-diol (retention time 7.613 min, m/z 243.1008) (Figure 4A). The identifications are tentative and were not confirmed by authentic standards or quantitative analysis.

The proximate analysis of whole plant *O. biflora* showed a moisture content of 3.31% ± 0.01%, crude fiber of 16.8%, crude protein of 2.86% ± 0.08%, and an ash content of 4.64% ± 0.04%. Total carbohydrates accounted for 60.32% of the dry weight, and total dietary fiber content was 65.1 g dry sample (Table 2). These values describe the nutritional composition of the whole plant material and do not represent the chemical composition of the hexane extract used in biological assays.

**TABLE 2:** Proximate composition and dietary fiber of *O. biflora*.

Parameters	Quantity
Carbohydrates	60.32%
Ash	4.64% ± 0.04%
Moisture	12.07% ± 0.03%
Fat	3.31% ± 0.01%
Crude protein	2.86% ± 0.08%
Crude fiber	16.8%
Dietary fiber	65.1 g

## Discussion

### Effect of the different extracts of *O. biflora* on the growth of *A. muciniphila in vitro*

The ability of the *Odontosoria biflora* hexane extract (OBE HEX) to promote *A. muciniphila* growth (Figure 2) suggests that this fern contains lipophilic or semi-polar metabolites capable of supporting bacterial proliferation under controlled anaerobic conditions. Similar growth-supporting effects have been reported for plant-derived phytochemicals in simplified culture systems, where nutrient availability and redox condition are optimized for bacterial metabolism (Xia et al., 2021; Rodríguez-Daza and de Vos, 2022). These findings suggest that the bioactive constituents of OBE-HEX may exert a prebiotic effect by selectively promoting the growth of *A. muciniphila*. Importantly, the *in vitro* assay was conducted under controlled culture conditions to identify extract fractions with direct growth-supporting activity toward *A. muciniphila*, and it is not intended to replicate the complexity of intestinal exposure *in vivo*. Therefore, the observed stimulation should be interpreted as an extract-dependent effect in a simplified system and does not, by itself, imply sustained enrichment or functional modulation of *A. muciniphila* within the gut.

### Acute oral toxicity assay

The acute oral toxicity assessment demonstrated that *O. biflora* hexane extract (OBE-HEX) produced no mortality, behavioral abnormalities, or adverse clinical signs up to 2000 mg/kg, indicating a wide safety margin in accordance with (OECD Test No. 423, 2002). The absence of toxic manifestations is consistent with findings from other medicinal plant extract such as *Clerodendrum viscosum* and *Leucas indica* (Chandrashekar et al., 2022). These results support the suitability of the extract for subsequent metabolic studies and justify the use of 250 and 500 mg/kg as biologically tolerable *in vivo* doses.

### Effect of OBE on fecal abundance of Akkermansia muciniphila

Fecal qPCR analysis demonstrated that *A. muciniphila* did not exhibit sustained enrichment in extract-treated groups despite pronounced growth stimulation observed *in vitro*. Rather than indicating lack of biological activity, this divergence underscores the context-dependent nature of microbial responses and highlights the limitations of extrapolating culture-based findings to the host gut environment, where ecological and host-mediated constraints govern bacterial persistence.

*In vitro*, the hexane fraction of *Odontosoria biflora* robustly promoted *A. muciniphila* growth in a dose-responsive manner, indicating the presence of bioactive lipophilic or semi-polar metabolites capable of directly supporting bacterial proliferation under controlled anaerobic conditions. Similar effects have been reported for plant-derived phytochemicals in simplified culture systems, where nutrient availability and redox conditions favor mucinolytic metabolism (Xia et al., 2021; Rodríguez-Daza and de Vos, 2022). In contrast, maintenance of *A. muciniphila in vivo* is influenced by additional factors such as mucin availability, epithelial turnover, host immune regulation, and microbial competition, which collectively shape colonization dynamics and limit sustained mono-taxon expansion (Cani and de Vos, 2017; Liu et al., 2024).

In the high-dose extract group (DM + HOBE), a transient increase in *A. muciniphila* qPCR signal was observed early during treatment, followed by a decline at later time points. This pattern suggests that OBE-derived metabolites may initially favor *A. muciniphila* detection under diabetic conditions but are insufficient to maintain prolonged enrichment. Similar transient responses have been reported following polyphenol-rich dietary interventions, where short-term increases in *A. muciniphila* do not necessarily translate into sustained colonization (Anhê et al., 2015; Jeong et al., 2020). Importantly, because no community-wide or functional microbiome analyses were performed, inferences regarding broader ecological restructuring cannot be drawn from the present data.

In contrast, the low-dose extract group (DM + LOBE) exhibited a progressive reduction in *A. muciniphila* qPCR signal, which may reflect insufficient exposure to bioactive metabolites to support detectable persistence under *in vivo* conditions. These findings emphasize that extract dose influences the magnitude and direction of *A. muciniphila*–specific responses, while also highlighting the sensitivity of fecal qPCR measurements to host and environmental factors in diabetic models.

The metformin-treated group showed sustained elevation of *A. muciniphila* by the final time point, consistent with prior reports linking metformin to enrichment of mucin-degrading bacteria through host-mediated mechanisms, including altered intestinal glucose flux and bile acid metabolism (Forslund et al., 2015; de la Cuesta-Zuluaga et al., 2017; Wu et al., 2017). This contrast further underscores that persistent *in vivo* enrichment of *A. muciniphila* is treatment-specific and not solely attributable to direct bacterial growth promotion observed *in vitro*.

Overall, these findings demonstrate that while *O. biflora* hexane extract strongly stimulates *A. muciniphila* growth under controlled *in vitro* conditions, its *in vivo* effects on fecal *A. muciniphila* detection are transient and context dependent. The observed qPCR trends reflect the complexity of host–microbe interactions rather than evidence of sustained enrichment or broad microbiota modulation. Accordingly, conclusions are limited to *A. muciniphila*–specific responses, and further studies incorporating community-level and functional analyses are required to determine whether *O. biflora* extract exerts wider microbiome or host-mediated metabolic effects.

### Phytochemical profiling and proximate analysis of O. biflora

The metabolites and proximate analysis of *Odontosoria biflora* provide a contextual information for interpreting the divergent *in vitro* and *in vivo* observations obtained in this study, while also highlighting important boundaries in data interpretation. Untargeted LC-MS analysis of the hexane extract yielded putative identification of two prominent metabolites, 2-O-rhamnosylvitexin (vitexin 2″-O-rhamnoside) and 7-methoxy-9,10-dihydrophenanthrene-2,5-diol (lusianthridin) based on spectral similarity to database entries. As these compounds were neither structurally confirmed nor quantified, the chemical analysis should be regarded as exploratory and hypothesis-generating rather than definitive.

Vitexin-2″-O-rhamnoside is a flavone C-glycoside commonly reported in plant extracts with microbiota-modulating potential, whereas lusianthridin belongs to the dihydrophenanthrene class of phenanthrenoids known for biological activity in microbial and host systems (Qi et al., 2021; Tang et al., 2024). Although no direct causal relationship between individual metabolites and *A*. *muciniphila* growth was established in the present study. The observed *in vitro* growth stimulation therefore reflects extract-level bioactivity under controlled culture conditions rather than the action of specific, validated compounds.

Importantly, the proximate composition data describe the nutritional characteristics of the whole *O. biflora* plant and are not directly applicable to the hexane extract evaluated *in vitro* and *in vivo*. In particular, dietary fiber and complex carbohydrates are unlikely to be present in the hexane fraction and should not be interpreted as contributors to the observed extract-dependent effects. Accordingly, biological responses associated with OBE-HEX are most plausibly attributed to lipophilic or semi-polar constituents detected by LC-MS, rather than to bulk nutritional components of the plant material.

The lack of sustained *in vivo* enrichment of *A. muciniphila*, despite pronounced *in vitro* growth stimulation, further underscores the context-dependent nature of microbial responses. While OBE-HEX demonstrated direct growth-supporting activity in culture, qPCR analysis revealed only transient changes in fecal *A. muciniphila* detection, consistent with known host-mediated and ecological constraints governing bacterial persistence in the gut environment (Cani and de Vos, 2017; Liu et al., 2024). Because no community-wide or functional microbiome analyses were performed, conclusions are limited to species-specific responses and do not support claims of broader microbiota modulation or ecological restructuring.

Taken together, these findings support a model in which *O. biflora* hexane extract exhibits extract-level bioactivity toward *A. muciniphila* under simplified *in vitro* conditions, while *in vivo* outcomes are constrained by host and environmental factors. This reinforces the importance of cautious interpretation of culture-based screening results and highlights the need for future studies incorporating targeted chemical validation, community-level microbiome analyses, and host metabolic endpoints.

### Conclusions

In conclusion, *Odontosoria biflora* hexane extract (OBE-HEX) is orally well tolerated and exhibits direct growth-supporting activity toward *A. muciniphila in vitro*, but this effect does not translate into sustained *in vivo* enrichment under diabetic conditions. The observed divergence underscores the limitations of extrapolating culture-based microbiota screening results to host-associated systems and highlights the role of host-dependent ecological constraints. Further studies integrating expanded microbiome analyses and targeted chemical validation are needed to clarify the biological relevance of these findings.

## Data Availability

The data generated during this study are available in Figshare at https://doi.org/10.6084/m9.figshare.31287289.

## Footnotes

1
http://prime.psc.riken.jp/compms/msdial/main.html
